# Supplementary material for: Epidemiology and Genetic Diversity of Spirometra Tapeworm Isolates from Snakes in Hunan Province, China
Source: Animals (Basel). 2022 May 9;12(9):1216. doi: 10.3390/ani12091216 (PMC9101633; doi:10.3390/ani12091216)
Supplement: Supplementary file 1 [file animals-12-01216-s001.zip › Table S1 forxml.pdf]

Table S1. Primers used to amplify the sequences studied.

Gene	Name	Sequence (5′–3′)	References
*cox*1	cox1F	TAGACTAAGTGTTTTCAAAACACTA	[37]
	cox1R	ATAGCATGATGCAAAAGG	
*cyt*b	cobF	TGATAGGTTATTTAAACTGGC	[37]
	cobR	TCAACAGTTGAAACAACCA	
