# Supplementary material for: Epidemiology and Genetic Diversity of Spirometra Tapeworm Isolates from Snakes in Hunan Province, China
Source: Animals (Basel). 2022 May 9;12(9):1216. doi: 10.3390/ani12091216 (PMC9101633; doi:10.3390/ani12091216)
Supplement: Supplementary file 1 [file animals-12-01216-s001.zip › Table S2 for xml.pdf]

Table S2. *Spirometra* isolates included in the molecular analysis.

**Country of origin**	**Host**	**Location**	**Sample codes**	**References**
China				
Yiyang City, Hunan	*Zaocys dhumnades*	112°46′ E, 28°59′ N	HuN-YiY1	This study
	Z. dhumnades	112°46′ E, 28°59′ N	HuN-YiY2	This study
	Z. dhumnades	112°46′ E, 28°59′ N	HuN-YiY3	This study
	Elaphe carinata	112°46′ E, 28°59′ N	HuN-YiY4	This study
	E. carinata	112°46′ E, 28°59′ N	HuN-YiY5	This study
Changde City, Hunan	*Z. dhumnades*	111°96′ E, 28°77′ N	HuN-CD2	This study
	Z. dhumnades	111°96′ E, 28°77′ N	HuN-CD3	This study
	E. carinata	111°96′ E, 28°77′ N	HuN-CD4	This study
	E. carinata	111°96′ E, 28°77′ N	HuN-CD5	This study
Yongzhou City, Hunan	*Z. dhumnades*	112°13′ E, 25°67′ N	HuN-YZ1	This study
	Z. dhumnades	112°13′ E, 25°67′ N	HuN-YZ2	This study
	Z. dhumnades	112°13′ E, 25°67′ N	HuN-YZ3	This study
Hengyang City, Hunan	*Z. dhumnades*	112°85′ E, 27°24′ N	HuN-HY1	This study
	Z. dhumnades	112°85′ E, 27°24′ N	HuN-HY2	This study
	Z. dhumnades	112°85′ E, 27°24′ N	HuN-HY3	This study
	E. carinata	112°85′ E, 27°24′ N	HuN-HY4	This study
	E. carinata	112°85′ E, 27°24′ N	HuN-HY5	This study
Xiangtan City, Hunan	*Z. dhumnades*	112°75′ E, 27°59′ N	HuN-XT1	This study
	Z. dhumnades	112°75′ E, 27°59′ N	HuN-XT2	This study
	Z. dhumnades	112°75′ E, 27°59′ N	HuN-XT3	This study
	E. carinata	112°75′ E, 27°59′ N	HuN-XT4	This study
	E. carinata	112°75′ E, 27°59′ N	HuN-XT5	This study
Shaoyang City, Hunan	*Z. dhumnades*	110°73′ E, 27°25′ N	HuN-SY1	This study

	Z. dhumnades	110°73′ E, 27°25′ N	HuN-SY2	This study
	Z. dhumnades	110°73′ E, 27°25′ N	HuN-SY3	This study
	E. carinata	110°73′ E, 27°25′ N	HuN-SY4	This study
	E. carinata	110°73′ E, 27°25′ N	HuN-SY5	This study
Zhuzhou City, Hunan	*Z. dhumnades*	113°43′ E, 26°61′N	HuN-ZZ1	This study
	Z. dhumnades	113°43′ E, 26°61′N	HuN-ZZ2	This study
	Z. dhumnades	113°43′ E, 26°61′N	HuN-ZZ3	This study
	Elaphe. taeniura	113°43′ E, 26°61′N	HuN-ZZ4	This study
	E. taeniura	113°43′ E, 26°61′N	HuN-ZZ5	This study
Changsha City, Hunan	*Z. dhumnades*	113°13′ E, 28°19′ N	HuN-CS1	This study
	Z. dhumnades	113°13′ E, 28°19′ N	HuN-CS2	This study
	Z. dhumnades	113°13′ E, 28°19′ N	HuN-CS3	This study
	White Tiger	113°01′ E, 28°04′ N	HuN-BT1	This study
	White Tiger	113°01′ E, 28°04′ N	HuN-BT2	This study
	White Tiger	113°01′ E, 28°04′ N	HuN-BT3	This study
	Panthera tigris	113°01′ E, 28°04′ N	HuN-DT1	This study
	Panthera tigris	113°01′ E, 28°04′ N	HuN-DT2	This study
	Panthera tigris	113°01′ E, 28°04′ N	HuN-DT3	This study
	Prionailurus bengalensis	113°01′ E, 28°04′ N	HuN-Li1	This study
	P. bengalensis	113°01′ E, 28°04′ N	HuN-Li2	This study
	P. bengalensis	113°01′ E, 28°04′ N	HuN-Li3	This study
	Cat	113°01′ E, 28°04′ N	HuN-C1	This study
	Cat	113°01′ E, 28°04′ N	HuN-C2	This study
	Cat	113°01′ E, 28°04′ N	HuN-C3	This study
Loudi City, Hunan	*E. carinata*	112°12′ E, 27°32′ N	HuN-LD1	This study
	E. carinata	112°12′ E, 27°32′ N	HuN-LD2	This study
	E. carinata	112°12′ E, 27°32′ N	HuN-LD3	This study
Chenzhou City, Hunan	*Z. dhumnades*	13°29′ E, 26°48′ N	HuN-CZ1	This study

	Z. dhumnades	13°29′ E, 26°48′ N	HuN-CZ2	This study
	Z. dhumnades	13°29′ E, 26°48′ N	HuN-CZ3	This study
Huaihua City, Hunan	*Z. dhumnades*	110°86′ E, 28°88′ N	HuN-HH1	This study
	Z. dhumnades	110°86′ E, 28°88′ N	HuN-HH2	This study
	Z. dhumnades	110°86′ E, 28°88′ N	HuN-HH3	This study
Zhangjiajie City, Hunan	*Z. dhumnades*	110°83′ E, 29°14′ N	HuN-ZZJ1	This study
	Z. dhumnades	110°83′ E, 29°14′ N	HuN-ZZJ2	This study
	Z. dhumnades	110°83′ E, 29°14′ N	HuN-ZZJ3	This study
Yueyang City, Hunan	*Z. dhumnades*	113°72′ E, 28°75′ N	HuN-YuY1	This study
	Z. dhumnades	113°72′ E, 28°75′ N	HuN-YuY2	This study
	Z. dhumnades	113°72′ E, 28°75′ N	HuN-YuY3	This study
	E. taeniura	113°72′ E, 28°75′ N	HuN-YuY4	This study
	E. taeniura	113°72′ E, 28°75′ N	HuN-YuY5	This study
Xiangxi City, Hunan	*Z. dhumnades*	109°54′ E, 29°09′ N	HuN-XX1	This study
	Z. dhumnades	109°54′ E, 29°09′ N	HuN-XX2	This study
	Z. dhumnades	109°54′ E, 29°09′ N	HuN-XX3	This study
Guilin City, Guangxi	*R. rugulosa*	110°2′ E, 25°23′ N	GX-GL-1	[13]
	R. rugulosa	110°2′ E, 25°23′ N	GX-GL-2	[13]
	R. rugulosa	110°2′ E, 25°23′ N	GX-GL-3	[13]
Nanning City, Guangxi	*E. chinensis*	108°48′E, 22°75′N	GX-NN-1	[13]
	E. chinensis	108°48′E, 22°75′N	GX-NN-2	[13]
	R. rugulosa	108°48′E, 22°75′N	GX-NN-3	[13]
	R. rugulosa	108°48′E, 22°75′N	GX-NN-4	[13]
	R. nigromaculata	108°48′E, 22°75′N	GX-NN-7	[13]
Yunlin city, Guangxi	*R. rugulosa*	110°27′E, 22°33′N	GX-YL-1	[13]
	R. rugulosa	110°27′E, 22°33′N	GX-YL-2	[13]
	R. nigromaculata	110°27′E, 22°33′N	GX-YL-5	[13]
	R. nigromaculata	110°27′E, 22°33′N	GX-YL-6	[13]
Haikou city, Hainan	*R. rugulosa*	110°32′E, 20°03′N	HN-HK-1	[13]
	R. rugulosa	110°32′E, 20°03′N	HN-HK-2	[13]
	R. nigromaculata	110°32′E, 20°03′N	HN-HK-4	[13]

Wuzhishan city, Hainan	*R. rugulosa*	110°4′E, 18°8′N	HN-WZS-1	[13]
	R. rugulosa	110°4′E, 18°8′N	HN-WZS-3	[13]
	R. nigromaculata	110°4′E, 18°8′N	HN-WZS-4	[13]
	R. nigromaculata	110°4′E, 18°8′N	HN-WZS-6	[13]
Guiyang city, Guizhou	*Enhydris chinensis*	106°63′E, 26°65′N	GZ-GY-1	[13]
	R. nigromaculata	106°63′E, 26°65′N	GZ-GY-4	[13]
Anshun city, Guizhou	*R. nigromaculata*	105°95′E, 26°25′N	GZ-AS-1	[13]
Jishou city, Hunan	*R. nigromaculata*	109°73′E, 28°32′N	HuN-JS-4	[13]
	R. nigromaculata	109°73′E, 28°32′N	HuN-JS-5	[13]
	R. nigromaculata	109°73′E, 28°32′N	HuN-JS-6	[13]
Changsha city, Hunan	*R. nigromaculata*	112°93′E, 28°23′N	HuN-CS-2	[13]
Guangan city, Sichuan	*R. nigromaculata*	106°11′E, 30°15′N	SC-GA-1	[36]
	R. nigromaculata	106°11′E, 30°15′N	SC-GA-2	[36]
	R. nigromaculata	106°11′E, 30°15′N	SC-GA-3	[36]
	R. nigromaculata	106°11′E, 30°15′N	SC-GA-5	[36]
Luzhou city, Sichuan	*R. nigromaculata*	105°55′E, 28°55′N	SC-LZ-1	[36]
	R. nigromaculata	105°55′E, 28°55′N	SC-LZ-2	[36]
	R. nigromaculata	105°55′E, 28°55′N	SC-LZ-4	[36]
	R. nigromaculata	105°55′E, 28°55′N	SC-LZ-5	[36]
Mianyang city, Sichuan	*R. nigromaculata*	104°35′E, 31°55′N	SC-MY-1	[36]
Nanchong city, Sichuan	*R. nigromaculata*	105°50′E, 30°35′N	SC-NC-1	[36]
	R. nigromaculata	105°50′E, 30°35′N	SC-NC-3	[36]
	R. nigromaculata	105°50′E, 30°35′N	SC-NC-4	[36]
	R. nigromaculata	105°50′E, 30°35′N	SC-NC-5	[36]
Baoshan city, Yunnan	*R. nigromaculata*	95°25′E, 100°02′N	YN-BS-1	[36]
	R. nigromaculata	95°25′E, 100°02′N	YN-BS-3	[36]
	R. nigromaculata	95°25′E, 100°02′N	YN-BS-4	[36]
Dehongzhou city, Yunnan	*R. nigromaculata*	97°31′E, 98°43′N	YN-DHZ-1	[36]
	R. nigromaculata	97°31′E, 98°43′N	YN-DHZ-2	[36]
Kunming city, Yunnan	*R. nigromaculata*	102°10′E, 24°23′N	YN-KM-1	[36]
	R. nigromaculata	102°10′E, 24°23′N	YN-KM-3	[36]
Liangping district, Chongqing	*R. nigromaculata*	107°80′E, 30°44′N	CQ-LP-1	[36]
	R. nigromaculata	107°80′E, 30°44′N	CQ-LP-3	[36]
Poland				
Białowieża Forest (BF)	*Nyctereutes procyonoides*	23°67′ E, 52° 89′ N	H01	[15]

	N. procyonoides	23°67′ E, 52° 89′ N	H02	[15]
	N. procyonoides	23°67′ E, 52° 89′ N	H03	[15]
	N. procyonoides	23°67′ E, 52° 89′ N	H04	[15]
	N. procyonoides	23°67′ E, 52° 89′ N	H05	[15]
	N. procyonoides	23°67′ E, 52° 89′ N	H06	[15]
	N. procyonoides	23°67′ E, 52° 89′ N	H07	[15]
	N. procyonoides	23°67′ E, 52° 89′ N	H10	[15]
	N. procyonoides	23°67′ E, 52° 89′ N	H11	[15]
	N. procyonoides	23°67′ E, 52° 89′ N	H12	[15]
	Meles meles	23°67′ E, 52° 89′ N	H13	[15]
	M. meles	23°67′ E, 52° 89′ N	H14	[15]
	M. meles	23°67′ E, 52° 89′ N	H15	[15]
	M. meles	23°67′ E, 52° 89′ N	H16	[15]
	M. meles	23°67′ E, 52° 89′ N	H18	[15]
	N. procyonoides	23°67′ E, 52° 89′ N	H23	[15]
	N. procyonoides	23°67′ E, 52° 89′ N	H24	[15]
	N. procyonoides	23°67′ E, 52° 89′ N	H25	[15]
	N. procyonoides	23°67′ E, 52° 89′ N	H26	[15]
	N. procyonoides	23°67′ E, 52° 89′ N	H27	[15]
	N. procyonoides	23°67′ E, 52° 89′ N	H28	[15]
	N. procyonoides	23°67′ E, 52° 89′ N	H29	[15]
	N. procyonoides	23°67′ E, 52° 89′ N	H30	[15]
	N. procyonoides	23°67′ E, 52° 89′ N	H31	[15]
	N. procyonoides	23°67′ E, 52° 89′ N	H32	[15]
	N. procyonoides	23°67′ E, 52° 89′ N	H33	[15]
	N. procyonoides	23°67′ E, 52° 89′ N	H34	[15]
	N. procyonoides	23°67′ E, 52° 89′ N	H37	[15]
	N. procyonoides	23°67′ E, 52° 89′ N	H38	[15]
	N. procyonoides	23°67′ E, 52° 89′ N	H39	[15]
	N. procyonoides	23°67′ E, 52° 89′ N	H40	[15]
	N. procyonoides	23°67′ E, 52° 89′ N	H41	[15]
	N. procyonoides	23°67′ E, 52° 89′ N	H42	[15]
	N. procyonoides	23°67′ E, 52° 89′ N	H43	[15]
	N. procyonoides	23°67′ E, 52° 89′ N	H44	[15]
	N. procyonoides	23°67′ E, 52° 89′ N	H45	[15]

	M. meles	23°67′ E, 52° 89′ N	H47	[15]
	M. meles	23°67′ E, 52° 89′ N	H49	[15]
	N. procyonoides	23°67′ E, 52° 89′ N	H50	[15]
	M. meles	22° 96′ E, 53° 80′ N	H58	[15]
	M. meles	22° 96′ E, 53° 80′ N	H59	[15]
	N. procyonoides	23°67′ E, 52° 89′ N	H61	[15]
	N. procyonoides	23°67′ E, 52° 89′ N	H62	[15]
	N. procyonoides	23°67′ E, 52° 89′ N	H63	[15]
	N. procyonoides	23°67′ E, 52° 89′ N	H64	[15]
	N. procyonoides	23°67′ E, 52° 89′ N	H65	[15]
	N. procyonoides	23°67′ E, 52° 89′ N	H66	[15]
	N. procyonoides	23°67′ E, 52° 89′ N	H67	[15]
	N. procyonoides	23°67′ E, 52° 89′ N	H68	[15]
	N. procyonoides	23°67′ E, 52° 89′ N	H69	[15]
	N. procyonoides	23°67′ E, 52° 89′ N	H70	[15]
	N. procyonoides	23°67′ E, 52° 89′ N	H71	[15]
	N. procyonoides	23°67′ E, 52° 89′ N	H72	[15]
	N. procyonoides	23°67′ E, 52° 89′ N	H73	[15]
	N. procyonoides	23°67′ E, 52° 89′ N	H74	[15]
	N. procyonoides	23°67′ E, 52° 89′ N	H75	[15]
	N. procyonoides	23°67′ E, 52° 89′ N	H76	[15]
	N. procyonoides	23°67′ E, 52° 89′ N	H77	[15]
	N. procyonoides	23°67′ E, 52° 89′ N	H78	[15]
	N. procyonoides	23°67′ E, 52° 89′ N	H79	[15]
Augustów Forest (AF)	*N. procyonoides*	22° 96′ E, 53° 80′ N	H08	[15]
	M. meles	22° 96′ E, 53° 80′ N	H09	[15]
	N. procyonoides	22° 96′ E, 53° 80′ N	H17	[15]
	M. meles	22° 96′ E, 53° 80′ N	H19	[15]
	M. meles	22° 96′ E, 53° 80′ N	H20	[15]
	M. meles	22° 96′ E, 53° 80′ N	H21	[15]
	N. procyonoides	22° 96′ E, 53° 80′ N	H22	[15]
	N. procyonoides	22° 96′ E, 53° 80′ N	H35	[15]
	N. procyonoides	22° 96′ E, 53° 80′ N	H36	[15]
	M. meles	22° 96′ E, 53° 80′ N	H46	[15]
	M. meles	22° 96′ E, 53° 80′ N	H48	[15]

	M. meles	22° 96′ E, 53° 80′ N	H51	[15]
	M. meles	22° 96′ E, 53° 80′ N	H52	[15]
	M. meles	22° 96′ E, 53° 80′ N	H53	[15]
	M. meles	22° 96′ E, 53° 80′ N	H54	[15]
	M. meles	22° 96′ E, 53° 80′ N	H55	[15]
	M. meles	22° 96′ E, 53° 80′ N	H56	[15]
	M. meles	22° 96′ E, 53° 80′ N	H57	[15]
	N. procyonoides	22° 96′ E, 53° 80′ N	H80	[15]
	N. procyonoides	22° 96′ E, 53° 80′ N	H81	[15]
Knyszyn Forest (KF)	*N. procyonoides*	23° 65′ E, 53° 26′ N	H60	[15]
	Mustela putorius	23° 65′ E, 53° 26′ N	H82	[15]
	M. putorius	23° 65′ E, 53° 26′ N	H83	[15]
	N. procyonoides	23° 65′ E, 53° 26′ N	H84	[15]
Masurian Lake District (MLD)	*N. procyonoides*	23° 65′ E, 53° 26′ N	H85	[15]
